# Determinants of favorable treatment outcomes in multidrug-resistant tuberculosis in Zambia: A retrospective cross sectional study, 2018–2022

**DOI:** 10.1371/journal.pone.0355721

**Published:** 2026-08-12

**Authors:** Dorcas Kalaba, Chitalu Chanda, Muhammad Mputu, Hamooya M. Benson, Matenge Mutalange, Lukundo Siame, Sepiso K. Masenga, Webster Chewe, Kaziwe Sikambale, Kabaso Mwewa, Uchizi Chirwa, David Mukube Sitali, Claude Mumba, Kamilo Mununga, Nancy Chanda-Kasese, Namakando Liusha, Kabaso Mushota, Watson Mtonga, Patrick Lungu, Angel Mubanga, Eustarckio Kazonga, Nkomba Kayeyi

**Affiliations:** 1 Department of Public Health, University of Lusaka (UNILUS), Lusaka, Zambia; 2 Department of Public Health, National TB and Leprosy Program, Lusaka, Zambia; 3 Department of Internal Medicine, University Teaching Hospital-Adult, Lusaka, Zambia; 4 Department of Public, Mulungushi University, Livingstone, Zambia; 5 Medical Department, AIDS Health Care Foundation, AHF, Lusaka, Zambia; 6 Department of Pharmacy, Marybegg Health Services, Ndola, Zambia; 7 Department of Internal Medicine, Levy Mwanawasa UTH, Lusaka, Zambia; 8 Department of Internal Medicine, Mopani Copper Mines, Mufulira, Zambia; 9 Department of Disease Control, School of Veterinary Medicine, University of Zambia (UNZA), Lusaka, Zambia; 10 Southern Africa Institute for Collaborative Research and Innovation Organisation (SAICRIO), Lusaka, Zambia; Freelance Consultant, Myanmar, MYANMAR

## Abstract

**Background:**

Multidrug-resistant tuberculosis (MDR-TB) is a persistent public health challenge in Zambia, with favorable treatment outcomes remaining below the World Health Organization target of 90%. However, limited national evidence on treatment outcomes and their determinants hinders the development of effective programmatic responses. This study aimed to estimate the proportion of MDR-TB patients achieving favorable treatment outcomes and to identify factors independently associated with these outcomes in Zambia from 2018 to 2022.

**Methods:**

A retrospective cross-sectional study was conducted using secondary data from the National Tuberculosis and Leprosy Programme Yathu database. All confirmed MDR-TB cases initiated on treatment across Zambia’s ten provinces from January 2018 to December 2022 were included. Demographic and clinical variables were extracted. Multivariable logistic regression was used to identify with factors independently associated with favorable treatment outcomes, reported as adjusted odds ratios (aORs) with 95% confidence intervals.

**Results:**

A total of 1,258 participants were included; the median age was 36 years (IQR: 28–44). Of these, 67.2% (n = 843) were male, while 53.0% (n = 662) were living with HIV. Overall, 68.1% (n = 857) achieved favorable treatment outcomes, while 31.9% (n = 401) had unfavorable outcomes. Normal body weight was positively associated with favorable outcomes (aOR 2.07; 95% CI: 1.02–4.21), while residing in Muchinga (aOR 0.12; 95% CI**:** 0.03–0.45; p = 0.002), and Western (aOR 0.17; 95% CI: 0.04–0.80; p = 0.024), was negatively associated with favorable outcomes.

**Conclusion:**

Favourable MDR-TB treatment outcomes in Zambia is shaped by nutritional status and marked geographic disparities. Strengthening nutritional support and targeting provincial inequities are essential to improving treatment outcomes.

## Introduction

Multidrug-resistant tuberculosis (MDR-TB), defined as resistance to at least isoniazid and rifampicin, the two most potent first-line anti-tuberculosis drugs, remains a critical threat to global public health [1]. In 2023, an estimated 410,000 new cases and 160,000 deaths were reported worldwide [2]. Globally, only 68% of MDR-TB patients achieve favorable treatment outcomes, falling well short of the 90% target established under the WHO End TB Strategy [2].

Sub-Saharan Africa bears a disproportionate share of this burden, accounting for approximately 25% of global MDR-TB cases. favorable treatment outcomes across the region remain persistently low, with studies from high-burden countries such as Ethiopia reporting outcomes well below global targets [3]. A systematic review and meta-analysis of MDR-TB and HIV co-infected patients across Sub-Saharan Africa similarly documented unfavorable and elevated mortality, underscoring the compounded challenge posed by the region’s dual epidemic of tuberculosis and HIV [4]. Zambia is classified by the WHO among the 30 high-burden countries for TB, HIV-associated TB, and MDR/RR-TB globally, a designation that reflects the severity of this dual epidemic within its borders [5]. Tuberculosis remains a leading cause of death among people living with HIV (PLHIV) in Zambia, driven by TB/HIV co-infection rates of 32% of all TB patients nationally [5]. Evidence from population-based analyses has further demonstrated that PLHIV in Zambia are significantly less likely to complete the TB care cascade compared to HIV-negative individuals, and that among those with rifampicin-resistant TB, only a fraction complete treatment, highlighting the urgent need for targeted interventions addressing both diseases simultaneously [6].

MDR-TB arises primarily through the inadequate or incomplete treatment of drug-susceptible tuberculosis, leading to the selection of resistant *Mycobacterium tuberculosis* strains, though direct transmission of resistant strains is increasingly documented in high-burden settings [5]. A consistent body of evidence identifies key clinical and sociodemographic determinants of unfavourable outcomes, including HIV co-infection, advanced age, low body mass index, malnutrition, extrapulmonary TB, prior TB treatment history, delayed treatment initiation, and poor adherence [7,8].

In Zambia, Monde et al. (2021) identified delayed specimen transport, inconsistent drug supply, and limited diagnostic access as significant programmatic barriers to MDR-TB care. Similarly, Chanda et al. (2024) reported that 21.3% of patients with drug-resistant tuberculosis died during treatment, with older age and male sex identified as significant predictors of mortality [9,10].

Despite this growing evidence base, significant gaps remain in understanding MDR-TB treatment outcomes at the national level in Zambia. Most existing studies are sub-national or facility based. Nanzaluka et al. (2019) drew from Lusaka only, Monde et al. (2021) from the Northern Region, Chanda et al. (2024) from the central region, and Siame et al. (2024) from a single hospital, limiting the generalisability of their findings to the broader national population [9–12]. Furthermore, gender-disaggregated outcome data are largely absent from publicly available programmatic reports, and no published study has systematically examined outcome trends across all provinces over multiple years using national programmatic data. Nasiri et al. (2025) highlighted this as a persistent “ceiling effect,” whereby gains demonstrated in clinical trials are not fully realised in operational settings due to unaddressed health system constraints [13].

This study, therefore, sought to address these evidence gaps by analysing programmatic data from Zambia’s National Tuberculosis and Leprosy Programme (NTLP) for the period 2018–2022. Specifically, it examined the proportion of MDR-TB patients achieving favourable treatment outcomes and identified the determinants associated with favorable treatment outcome. The findings aim to provide national-level evidence to guide equitable and effective drug-resistant TB policy and programming in Zambia.

## Methods

### Study design and setting

A retrospective cross sectional study was conducted using secondary data from Zambia’s National Tuberculosis and Leprosy Programme (NTLP) to evaluate treatment outcomes and associated factors among patients with multidrug-resistant tuberculosis (MDR-TB) between January 2018 and December 2022. The study included all MDR-TB treatment sites across Zambia’s ten provinces, encompassing primary, secondary, and tertiary healthcare facilities.

### Study population

The study population comprised all patients with laboratory-confirmed MDR-TB who were initiated on treatment between 1 January 2018 and 31 December 2022.

### Sample size and sampling

**Fig 1:**
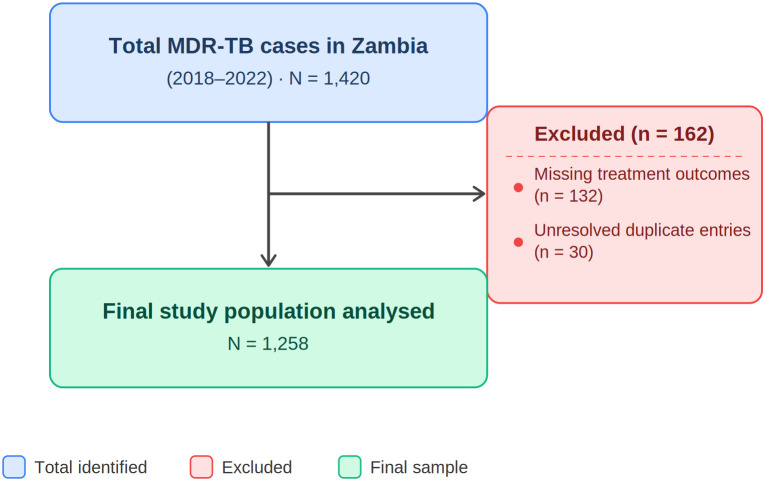
Study flow diagram showing inclusion and exclusion of participants.

A census sampling approach was used, including all patients with multidrug-resistant tuberculosis (MDR-TB) recorded in the National Tuberculosis and Leprosy Programme (NTLP) Yathu database between 1 January 2018 and 31 December 2022. No formal sample size calculation was performed. A total of 1,420 records were identified. After applying eligibility criteria, 162 records were excluded due to missing treatment outcomes (n = 132), and unresolved duplicate entries (n = 30). The final analytic sample comprised 1,258 patients with complete treatment outcome data (Fig 1).

### Variables

The primary outcome variable was treatment outcome, categorized as favorable (cured and treatment completed) and unfavorable (treatment failure, loss to follow-up, and death). Independent variables included demographic characteristics (age, sex, and province of residence), clinical and laboratory factors (HIV status, body mass index [BMI], baseline smear and culture results, drug susceptibility testing results, diagnostic modality, comorbidities such as diabetes mellitus, hypertension, chronic kidney disease, chronic respiratory disease, extrapulmonary tuberculosis, and behavioral factors including alcohol use and smoking), and programmatic factors (treatment regimen type, year of treatment initiation, and patient category).

### Operation definitions

1. Multidrug-resistant tuberculosis (MDR-TB) was defined as tuberculosis caused by *Mycobacterium tuberculosis* strains resistant to at least isoniazid and rifampicin, confirmed by molecular or phenotypic drug susceptibility testing, including the GeneXpert MTB/RIF assay for detection of rifampicin resistance, in accordance with World Health Organization and national tuberculosis programme guidelines [14,15].
2. Body Mass Index (BMI) was calculated as weight in kilograms divided by height in meters squared (kg/m^2^), recorded at treatment initiation. BMI was categorized according to World Health Organization (WHO) criteria as follows: Underweight: < 18.5 kg/m^2^, Normal weight: 18.5–24.9 kg/m^2^, Overweight/Obese: ≥ 25.0 kg/m^2^ [16].
3. Treatment outcomes were defined according to national guidelines. Favorable outcomes included patients who were cured or completed treatment [17]. Cure was defined as bacteriologically confirmed pulmonary TB with documented response to treatment and no evidence of failure, while treatment completion referred to patients who completed therapy without meeting criteria for cure or failure [17]. Unfavorable outcomes included treatment failure, lost to follow-up, or death [17]. Death was defined as death from any cause before or during treatment, and lost to follow-up as treatment interruption for two consecutive months or more [17].

### Data sources and management

Data was extracted from the NTLP’s Yathu database (the national DR-TB treatment monitoring system). Deduplication was conducted using unique patient identifiers (national patient ID and facility TB number). Data quality checks included range and logic checks for numerical variables, consistency checks across related fields (e.g., regimen start and end dates), and verification of outcome definitions in line with WHO and NTLP standards. All patient identifiers were removed before analysis; de-identified datasets were stored in encrypted, access-controlled folders.

### Data analysis

All analyses were conducted using Stata version 15.0 (StataCorp LLC, College Station, TX, USA). Categorical variables were summarised using frequencies and percentages, while continuous variables were described using medians and interquartile ranges (IQRs) due to non-normal distribution. Univariable logistic regression was performed to assess associations between independent variables and favorable treatment outcomes. Variables were selected for inclusion in the multivariable model using a two-step approach. First, variables with p < 0.20 in univariable analysis were eligible for inclusion. Second, additional variables were included regardless of univariable p-value if they were considered clinically or epidemiologically important a priori, based on established evidence from the MDR-TB literature. Prior to multivariable modelling, the assumption of linearity between age and the log odds of the outcome was assessed using the Box–Tidwell test (p = 0.137). The assumption was satisfied, and age was retained as a continuous linear term in the multivariable model. Multivariable analyses were performed using complete-case analysis, including only participants with complete data for all variables included in the final model. Results are presented as adjusted odds ratios (aORs) with 95% confidence intervals, and statistical significance was set at p < 0.05. Model fitness was assessed using the Hosmer-Lemeshow goodness-of-fit test (χ² = 3.164, df = 8, p = 0.924). Multicollinearity among independent variables was evaluated using variance inflation factors (VIF) (mean = 1.93, minimum = 1.03, maximum = 5.41).

### Ethical considerations

This study used secondary, de-identified programmatic data and involved no direct patient contact, posing minimal risk to participants. Patient anonymity and confidentiality were strictly maintained, with no identifiers linked to individual records. Ethical approval was obtained from the University of Lusaka Research Ethics Committee (FWA00033228–51008/25) on 1 December 2025 and the National Health Research Authority (NHRA-2987/04/12/2025) on 18 December 2025. Data collection was conducted from 3 January to 3 February 2026. Additional authorization to access and use the data was granted by the Ministry of Health and the National Tuberculosis and Leprosy Programme through a formal data use agreement. This study was reported in accordance with the STROBE Statement checklist for observational studies (S1 File).

## Results

A total of 1,258 participants were included, 857 (68.1%) achieved a favorable treatment outcome, while 401 (31.9%) had an unfavorable outcome. The median age of 36 years (IQR: 28–44). The majority were male (843/1,255; 67.2%) and predominantly from Lusaka Province (372/1,258; 29.6%), followed by Copperbelt (328/1,258; 26.1%). Among participants with available data, 53.8% (463/860) were married. Most had attained at least secondary education, with 27.1% (95/351) completing upper secondary level. The most common occupations were businesspersons (23.1%) and subsistence farmers (15.1%).

Alcohol consumption was reported by 42.0% (303/721) of participants, while 25.8% (185/736) reported smoking. Overall, 68.1% (857/1,258) of participants achieved favorable outcomes. Favorable outcomes were more common among participants who did not consume alcohol compared to those who consumed alcohol (50.2% vs. 38.5%, *p* = **0.003**) (see Table 1).

**Table 1:** Association between demographic and lifestyle characteristics and favorable MDR-TB treatment outcome, Zambia, 2018–2022.

Variable	n	Median (IQR) or n (%)	Favorable: Yes, 857 (68.1%)	Favorable: No, 401 (31.9%)	*p*-value
**Age**	1,258	36 (28, 44)	37 (29, 44)	35 (28, 45)	0.917
**Sex**	1,255				0.071
*Male*		843 (67.2)	561 (65.5)	282 (70.7)	
*Female*		412 (32.8)	295 (34.5)	119 (29.8)	
**Province**	1,258				**<0.001**
*Central*		122 (9.7)	90 (10.5)	32 (8.0)	
*Copperbelt*		328 (26.1)	221 (25.8)	107 (26.7)	
*Eastern*		69 (5.5)	48 (5.6)	21 (5.2)	
*Luapula*		86 (6.8)	59 (6.9)	27 (6.7)	
*Lusaka*		372 (29.6)	279 (32.6)	93 (23.2)	
*Muchinga*		29 (2.3)	10 (1.2)	19 (4.7)	
*North Western*		55 (4.4)	35 (4.1)	20 (5.0)	
*Northern*		56 (4.5)	30 (3.5)	26 (6.5)	
*Southern*		84 (6.7)	60 (7.0)	24 (6.0)	
*Western*		57 (4.5)	25 (2.9)	32 (8.0)	
**Marital status**	860				0.150
*Single*		318 (37.0)	212 (34.9)	106 (42.1)	
*Married*		463 (53.8)	340 (55.9)	123 (48.8)	
*Divorced*		55 (6.4)	37 (6.1)	18 (7.1)	
*Widowed*		24 (2.8)	19 (3.1)	5 (2.0)	
**Education**	351				0.806
*No education*		34 (9.7)	21 (8.7)	13 (11.8)	
*Lower primary*		73 (20.8)	53 (22.0)	20 (18.2)	
*Upper primary*		53 (15.1)	34 (14.1)	19 (17.3)	
*Lower secondary*		50 (14.3)	33 (13.6)	17 (15.5)	
*Upper secondary*		95 (27.1)	67 (27.8)	29 (25.5)	
*Tertiary*		46 (13.1)	33 (13.7)	13 (11.8)	
**Occupation**	662				0.554
*Public bus operator*		17 (2.6)	9 (1.9)	8 (4.2)	
*Construction worker*		10 (1.5)	27 (5.8)	12 (6.2)	
*Business person*		153 (23.1)	13 (2.8)	6 (3.1)	
*Domestic worker*		27 (4.1)	6 (1.3)	4 (2.1)	
*Miner/Ex-miner*		12 (1.8)	115 (24.5)	38 (19.7)	
*Health care worker*		11 (1.7)	21 (4.5)	6 (3.1)	
*Subsistence farmer*		100 (15.1)	8 (1.7)	4 (2.1)	
*Teacher/Lecturer*		10 (1.5)	9 (1.9)	2 (1.0)	
*Student*		12 (1.8)	72 (15.4)	28 (14.5)	
*Pupil*		39 (5.9)	7 (1.5)	3 (1.6)	
*Inmate*		19 (2.9)	11 (2.4)	1 (0.5)	
*Other*		252 (38.1)	171 (36.5)	81 (42.0)	
**Alcohol use**	721				**0.003**
*Yes*		303 (42.0)	194 (38.5)	109 (50.2)	
*No*		418 (58.0)	310 (61.5)	108 (49.8)	
**Smoking**	736				0.115
*Yes*		185 (25.8)	120 (23.4)	65 (29.3)	
*No*		532 (75.2)	378 (73.5)	154 (69.4)	

Note: Values are median (IQR) for continuous variables and n (%) for categorical variables. p-values derived from chi-square test for categorical variables and Mann-Whitney U test for age. Bold p-values indicate statistical significance (p < 0.05). IQR = interquartile range.

### Clinical characteristics

Most participants were new patients on treatment (872/1,250; 69.8%). A previous diagnosis was reported in 38.8% (440/1,135) of participants. Over half were living with HIV (662/1,250; 53.0%). Hypertension was present in 2.2% (28/1,250) of participants. Primary DRTB accounted for 64.5% (757/1,173) of cases. Most diagnoses were made at health facilities (1,178/1,250; 94.2%), and nearly all cases were pulmonary TB (1,225/1,243; 98.6%). Most participants were initiated on the short treatment regimen (596/1,004; 59.3%).

Most participants with normal body weight (BMI) had a favorable outcome compared to those who were underweight, overweight, or obese (56.7% vs 27.2% vs 11.1% vs 5.1%). Participants living without diabetes had a favorable outcome compared to those living with diabetes (99.3% vs 0.7%). Participants on the short-term TB regimen had a favorable outcome compared to those on the long-term TB regime (53.8% vs 46.3%) (see Table 2).

**Table 2:** Association between clinical characteristics and favorable outcome 2018–2022.

Variable	n	n (%)	Favorable: Yes, 857 (68.1%)	Favorable: No, 401 (31.9%)	*p*-value
**Type of patient**	1,250				0.065
*New*		872 (69.8)	597 (69.7)	275 (69.0)	
*Retreatment*		249 (19.9)	181 (21.1)	68 (17.3)	
*After LTFU*		15 (1.2)	7 (0.8)	8 (2.0)	
*Treatment after failure*		58 (4.6)	41 (4.8)	17 (4.3)	
*Previously treated, unknown outcome*		36 (2.9)	21 (2.5)	15 (3.8)	
*Trans-in*		20 (1.6)	10 (1.2)	10 (2.5)	
**Previous history of TB**	1,135				0.234
*Yes*		440 (38.8)	316 (40.0)	124 (36.1)	
*No*		695 (61.2)	475 (60.1)	220 (64.0)	
**HIV status**	1,250				0.437
*Positive*		662 (53.0)	447 (54.2)	215 (56.6)	
*Negative*		543 (43.4)	378 (45.1)	165 (43.4)	
**Baseline BMI**	459				**<0.001**
*Underweight*		151 (32.9)	86 (27.2)	65 (45.5)	
*Normal weight*		245 (53.4)	179 (56.7)	66 (46.2)	
*Overweight*		43 (9.4)	35 (11.1)	8 (5.6)	
*Obese*		20 (4.4)	16 (5.1)	4 (2.8)	
**Diabetes mellitus**	1,250				**0.037**
*Yes*		14 (1.1)	6 (0.7)	8 (2.0)	
*No*		1,236 (98.9)	851 (99.3)	385 (98.0)	
**Hypertension**	1,250				0.935
*Yes*		28 (2.2)	19 (2.2)	9 (2.3)	
*No*		1,222 (97.8)	838 (97.8)	384 (97.7)	
**Chronic kidney disease**	1,250				0.423
*Yes*		4 (0.3)	2 (0.2)	2 (0.5)	
*No*		1,246 (99.7)	855 (99.8)	391 (99.5)	
**COPD/Asthma**	1,250				0.072
*Yes*		7 (0.6)	7 (0.8)	0 (0.0)	
*No*		1,243 (99.4)	850 (99.2)	393 (100.0)	
**Type of DR-TB**	1,173				0.644
*Primary*		757 (64.5)	516 (64.1)	241 (65.5)	
*Secondary*		416 (35.6)	289 (35.9)	127 (34.5)	
**Place of diagnosis**	1,250				0.379
*Health facility*		1,178 (94.2)	811 (94.6)	367 (93.4)	
*Community*		72 (5.8)	46 (5.4)	26 (6.6)	
**TB classification**	1,243				0.757
*Pulmonary*		1,225 (98.6)	843 (98.5)	382 (98.7)	
*Extrapulmonary*		18 (1.5)	13 (1.5)	5 (1.3)	
**TB regimen**	1,004				**0.013**
*Short (≤12 months)*		596 (59.3)	424 (62.0)	172 (53.8)	
*Long (18 months)*		408 (40.7)	260 (38.0)	148 (46.3)	

Note: Values are n (%). p-values derived from chi-square test. Bold p-values indicate statistical significance (p < 0.05). BMI = body mass index; CKD = chronic kidney disease; COPD = chronic obstructive pulmonary disease; DR-TB = drug-resistant tuberculosis; LTFU = lost to follow-up; TB = tuberculosis.

### Regression analysis of factors associated with favorable (Cured and completed treatment) outcome

In the multivariable analysis, participants from Muchinga (aOR = 0.12; 95% CI: 0.03–0.45; p = 0.002) and Western provinces (aOR = 0.17; 95% CI: 0.04–0.80; p = 0.024) had significantly lower odds of achieving a favorable treatment outcome than those from Lusaka Province. In contrast, participants with normal BMI had more than twice the odds of a favorable treatment outcome compared with underweight participants (aOR = 2.14; 95% CI: 1.04–4.37; p = 0.038) (see Table 3).

**Table 3:** Univariable and multivariable logistic regression analysis of factors associated with favorable MDR-TB treatment outcome, Zambia, 2018–2022.

Variable	n	cOR	95% CI	p-value	aOR	95% CI	p-value
**Demographic**							
Age (per year increase)	1,258	1.00	0.99-1.00	0.626	1.01	0.98–1.04	0.391
**Sex**							
*Male*		*Ref*			*Ref*	–	–
Female	1,255	1.27	0.98-1.64	0.071	0.87	0.39–1.94	0.728
**Alcohol use**							
*Yes*		*Ref*			*Ref*	–	–
No	721	**1.61**	1.17-2.22	**0.004**	1.59	0.67–3.73	0.291
**Smoking**							
*Yes (Ref)*		*Ref*			*Ref*		
No	717	1.33	0.93–1.90	0.116	0.73	0.27–1.99	0.542
**BMI**							
*Underweight*		*Ref*			*Ref*	–	–
Normal weight	459	**2.05**	1.34–3.15	**0.001**	**2.14**	1.04–4.37	**0.038**
Overweight	459	**3.31**	1.44–7.61	**0.005**	3.10	0.61–15.74	0.172
Obese	459	3.02	0.96–9.47	0.058	7.50	0.55–101.30	0.129
**Province**							
*Lusaka*		*Ref*			*Ref*		
Central	1,258	0.94	0.59–1.50	0.786	–	–	–
Copperbelt	1,258	**0.69**	0.50–0.96	**0.026**	0.35	0.06–1.97	0.236
Eastern	1,258	0.76	0.43–1.34	0.345	1.15	0.32–4.15	0.835
Luapula	1,258	0.73	0.44–1.22	0.225	0.42	0.13–1.38	0.151
Muchinga	1,258	**0.18**	0.08–0.39	**<0.001**	**0.12**	0.03–0.45	**0.002**
North Western	1,258	0.58	0.32–1.06	0.077	0.42	0.03–6.43	0.535
Northern	1,258	**0.38**	0.22–0.68	**0.001**	0.32	0.08–1.23	0.098
Southern	1,258	0.83	0.49–1.41	0.499	0.75	0.29–1.97	0.561
Western	1,258	**0.26**	0.15–0.46	**<0.001**	**0.17**	0.04–0.80	**0.024**
**Type of patient**							
*New*		*Ref*			*Ref*		
Retreatment	1,250	1.23	0.90–1.68	0.202	0.64	0.17–2.37	0.502
After LTFU	1,250	0.40	0.14–1.12	0.082	0.26	0.03–2.56	0.249
Treatment after failure	1,250	1.11	0.62–1.99	0.724	0.30	0.05–1.69	0.171
Previously treated, unknown outcome	1,250	0.64	0.33–1.27	0.205	–		–
Trans-in	1,250	0.46	0.19–1.12	0.087	–		–
**Previous history of TB**							
*Yes*		*Ref*			*Ref*		
No	1,135	0.85	0.65–1.10	0.215	0.41	0.10–1.68	0.216
**HIV status**							
*Positive*		*Ref*			*Ref*		
Negative	1,205	1.10	0.86–1.41	0.437	1.61	0.81–3.22	0.178
**Type of DR-TB**							
*Primary*		*Ref*			*Ref*		
Secondary	1,173	1.06	0.82–1.38	0.644	0.64	0.15–2.70	0.545
**TB classification**							
*Pulmonary*		*Ref*			*Ref*		
Extrapulmonary	1,243	1.18	0.42–3.33	0.757	–		–
**TB regimen**							
*Short (≤12 months)*		*Ref*			*Ref*		
Long (18 months)	1,004	**0.71**	0.54–0.93	**0.013**	0.60	0.29–1.23	0.165

## Discussion

This study examined favorable MDR-TB treatment outcomes and their associated factors among 1,258 patients enrolled in Zambia’s National Tuberculosis and Leprosy Programme (NTLP) between 2018 and 2022. The overall treatment favorable rate of 68.1% observed in this study falls below WHO’s 90% target but is broadly consistent with regional and global benchmarks. Globally, approximately 68% of MDR-TB patients achieve favourable treatment outcome, and rates across Sub-Saharan Africa have been similarly reported to range from 45–65% [18]). A systematic review and meta-analysis of tuberculosis treatment outcomes across Africa by Teferi et al. (2021) reported a pooled favorable rate of approximately 72%, against which the present findings are contextually comparable [19]. In Zambia specifically, national favorable outcome rates have historically trailed the WHO target, a pattern attributable to the compounding effects of HIV co-infection, health system constraints, and geographic inaccessibility of services [6,9].

One of the key findings of this study was the independent association between normal body weight and favorable treatment outcomes. This observation is consistent with a substantial body of evidence identifying malnutrition and low body mass index (BMI) as important determinants of poor multidrug-resistant tuberculosis (MDR-TB) outcomes. Underweight BMI has been reported as an independent factor associated with unfavorable treatment outcomes, attributed to impaired immune function, reduced drug bioavailability, and diminished physiological resilience during the prolonged treatment course [7]. Similarly, nutritional deficiency has been identified as a strong factors associated with treatment failure in Northwest Ethiopia [8]. The biological mechanisms underpinning this relationship are well established. Malnutrition compromises cell mediated immunity, impairs gastrointestinal absorption of anti-tuberculosis drugs, and increases susceptibility to opportunistic infections, all of which hinder treatment response [7]. In the Zambian context, where food insecurity remains prevalent particularly in rural and peri urban settings, these findings carry important programmatic implications suggesting that integrating routine nutritional assessment and targeted supplementation into MDR-TB care may improve treatment outcomes and that nutritional support should be institutionalised as a core component of comprehensive MDR-TB management rather than treated as an adjunct intervention [20,21].

Marked geographic disparities in treatment outcomes were identified, with patients from Muchinga and Western provinces demonstrating lower likelihood of favorable outcomes relative to Lusaka. These provinces are predominantly rural and face well-documented health system constraints, including shortages of trained healthcare personnel, limited specialised MDR-TB treatment centres, chronic drug supply interruptions, weak referral systems, poor road infrastructure, and inadequate community-based treatment support, all. Programmatic evidence from Zambia supports this interpretation, where delays in specimen transport, inconsistent drug supply, and limited diagnostic capacity have been documented as key barriers to effective MDR-TB care [9]. The geographic patterns observed are consistent with broader evidence from sub-Saharan Africa, where provincial and district-level disparities in MDR-TB outcomes are often driven more by inequities in access to care than by differences in patient-level clinical characteristics [19]. Similarly, research from rural South Africa has shown that geographic remoteness can act as a major barrier to treatment continuity and adherence, contributing to poorer outcomes [22]. These findings highlight the need for targeted investments in decentralized MDR-TB service delivery, expansion of mobile treatment support, and province-specific health system strengthening strategies to address persistent geographic inequities.

Contrary to widely held assumptions and prior evidence from sub-Saharan Africa, HIV co-infection was not independently associated with unfavorable treatment outcomes in this study. Although HIV remains a major driver of the tuberculosis epidemic in Zambia (Lungu et al., 2021; World Health Organization, 2025), this finding is consistent with emerging evidence from high-burden settings where antiretroviral therapy (ART) scale-up has substantially reduced the negative impact of HIV on treatment outcomes [5,6]. In South Africa, concurrent treatment of MDR-TB and HIV with ART has been shown to improve survival and cure rates among co-infected patients to levels comparable with HIV-negative individuals [23]. Similarly, findings from a rural high HIV-burden setting demonstrated that integration of ART with standardised MDR-TB regimens narrowed disparities in outcomes between HIV-positive and HIV-negative patients [24]. Zambia’s substantial investment in ART coverage, with a high proportion of people living with HIV receiving treatment (World Health Organization, 2025), may therefore contribute to mitigating the historically adverse impact of HIV on MDR-TB outcomes [5]. However, this finding should be interpreted with caution, given the high proportion of missing data on ART status, which may have introduced residual confounding.

Regimen type, whether short all-oral or longer individualised treatment, was not independently associated with favorable outcomes in this study. This finding aligns with an emerging body of evidence suggesting that both short and long MDR-TB regimens can achieve comparable success rates when implemented under adequate programmatic conditions [25]. Increasingly, evidence indicates that treatment effectiveness in real-world settings is shaped less by regimen duration and more by health system factors such as adherence support, quality of patient monitoring, and consistent drug availability [13]. In a 15-year trend analysis of drug-resistant pulmonary tuberculosis, Nasiri et al. (2025) highlighted a persistent “ceiling effect” in operational settings, whereby improvements demonstrated in clinical trials are not fully translated into routine programmatic effectiveness due to unresolved health system constraints [13]. This pattern is particularly relevant in the Zambian context, where structural and programmatic limitations may continue to influence MDR-TB treatment outcomes irrespective of regimen type.

Sex and prior treatment history were not independently associated with treatment outcomes in this study. Although males constituted the majority of the cohort, this pattern is consistent with the well-documented male predominance of tuberculosis globally and within Zambia [18]. The absence of a statistically significant association between sex and treatment outcomes may reflect limited power to detect subgroup differences or a true attenuation of sex-related effects after adjustment for confounding factors. Nonetheless, this finding remains important and warrants further investigation using gender-disaggregated analyses, as differences in health-seeking behaviour, access to care, and social support structures may influence treatment outcomes in ways that are not fully captured in aggregate analyses.

Alcohol consumption was not independently associated with treatment outcomes in this study after adjustment for potential confounders, suggesting that the observed relationship may be mediated through underlying sociodemographic and clinical factors. Nevertheless, the high prevalence of alcohol use within the cohort remains clinically significant, as alcohol consumption has been consistently linked to poor treatment adherence, adverse drug interactions, and increased risk of hepatotoxicity during multidrug-resistant tuberculosis (MDR-TB) therapy. These factors can indirectly compromise treatment effectiveness and patient safety, even in the absence of a direct independent association with outcomes [26]. This underscores the importance of incorporating structured alcohol screening and brief intervention strategies into MDR-TB care. Integrating such approaches within routine clinical management may support adherence, reduce treatment-related complications, and contribute to improved overall patient outcomes through a more comprehensive, patient-centered model of care.

This study has several important strengths. It draws on five years of national programmatic data, representing one of the most comprehensive population-level analyses of multidrug-resistant tuberculosis (MDR-TB) treatment outcomes in Zambia to date. Previous studies have largely been limited to subnational settings, including Lusaka (Nanzaluka et al., 2019), the Northern Region (Monde et al., 2021), Central Province (Chanda, 2024), and single-facility analyses (Siame et al., 2024), and the present study addresses this critical gap in national evidence [9–11]. The large sample size and use of the National Tuberculosis and Leprosy Programme (NTLP) Yathu database enhance the analytical robustness and generalisability of the findings. Nevertheless, several limitations should be acknowledged. As a retrospective secondary data analysis, the study is inherently dependent on the completeness and quality of routinely collected programmatic data. Missing data for key variables, including body mass index (BMI), antiretroviral therapy (ART) status, and other clinical characteristics, may have introduced selection bias and residual confounding. In addition, the very low prevalence of diabetes mellitus limited its evaluation in the multivariable analysis, and its independent association with treatment outcomes could not be reliably assessed. The observational nature of the study precludes causal inference. Although low BMI was associated with unfavorable treatment outcomes, the temporal relationship could not be established, and reverse causality cannot be excluded. Similarly, the interpretation of HIV-related findings should be made with caution because information on ART use, viral suppression, and immunological status was unavailable. Furthermore, the inability to assess time-to-event outcomes, including the timing and causes of mortality, limits the depth of epidemiological inference. Future prospective studies incorporating survival analysis and time-varying covariates are needed to provide a more nuanced understanding of MDR-TB treatment dynamics in this setting.

## Conclusion

This study provides comprehensive national-level evidence on multidrug-resistant tuberculosis (MDR-TB) treatment outcomes in Zambia and shows that favorable treatment outcome remain below targets set by the World Health Organization. Nutritional status and geographic location emerged as key determinants of outcomes, with better outcomes among patients of normal body weight and poorer outcomes in certain provinces, reflecting underlying health system disparities. These findings highlight the need to integrate nutritional support into routine MDR-TB care and to address provincial inequities through strengthened, decentralized services and improved patient support systems. Future research should focus on prospective designs and survival analyses to better understand outcome dynamics and inform targeted, equity-focused MDR-TB interventions in Zambia

## Supporting information

S1 File Strobe check list. (DOCX)

S2 File Minimal dataset. (XLSX)
