# Supplementary material for: Determinants of favorable treatment outcomes in multidrug-resistant tuberculosis in Zambia: A retrospective cross sectional study, 2018–2022
Source: PLoS One. 2026 Aug 12;21(8):e0355721. doi: 10.1371/journal.pone.0355721 (PMC13466015; doi:10.1371/journal.pone.0355721)
Supplement: Supplementary file 2 [file pone.0355721.s003.docx]

**Manuscript Number:** PONE-D-26-21768

**Title:** Determinants of Favorable Treatment Outcomes in Multidrug-Resistant Tuberculosis in Zambia: A Retrospective Cohort Study, 2018-2022

**Recommendation:** Major Revision

This is a highly valuable, large-scale retrospective cohort analysis leveraging Zambia's national NTLP Yathu registry to uncover vital geographic and clinical disparities in MDR-TB care success.

However, before publication can be recommended, the following major and minor concerns must be resolved:

1. **Introduction**

Line 76: species name - write in italic

Line 83-85: the text ‘while Bwembya

84 et al. (2024) found that 75% of TB-related deaths occurred within the first two months of treatment,85 predominantly among HIV-positive individuals’ change to research focusing on MDR TB.

2. **Methods**

**Figure 1:** Please re-upload or fix Figure 1 (the study flow diagram), as it fails to display clearly the research flow.

Line 132: remove one post stop.

Line 140: Operational definition

- How about weight? The weight at which the month of treatment was used?

- **Line 147–149:** **Mortality** was defined as death from any cause occurring before or during the course of anti-tuberculosis treatment..."

**Line 160–162:** **Death** was defined as death from any cause before or during treatment..."

Operational definitions no2 (Mortality) and no4 (under Unfavorable outcomes) are entirely redundant and identical in substance. Please consolidate these into a single definition to streamline the Methods section.

Line 143: italic for species name ‘*Mycobacterium tuberculosis”*

3. **Results**

The study aims (Line 98-99) stated to examine the % of MDR. However, no results were presented in the text.

Line 206: capital T for Table 1

Table 1 & 2 & 3: in the last column, showing p-value. What analysis was used? In the text above, nothing was mentioned about the p-value. So, is the analysis necessary? The table should be presented scientifically, i.e., without visible lines for rows and columns, except the uppermost and lowermost. Standardise in writing p, italic or not, small or capital letter. For example, line 179 and line 182. 95CI should be written completely, 95% CI.

Line 221: Results of the regression analysis should include the model fitness as stated in lines 182-185.

4. **Discussion**

Line 234: treatment success or favourable treatment? should be clear and standardised. Not only in this line, but throughout this manuscript.

Line 246-247: should be cautious in interpreting the results from a cross-sectional study. The big limitation is that it cannot explain the causal effects of the factors. Thus, for the BMI, which one is the predictor? Poor treatment outcome causes low BMI, or Low BMI causes poor treatment outcome?

Line 260-261: Elaborate on health care and support systems disparities among these provinces.

Line 288-289: also cautious on the interpretation since no information regarding Without knowing the exact proportion of PLHIV who were actively on ART, virally suppressed, or severely immunosuppressed (CD4 counts),
