# Supplementary material for: Determinants of favorable treatment outcomes in multidrug-resistant tuberculosis in Zambia: A retrospective cross sectional study, 2018–2022
Source: PLoS One. 2026 Aug 12;21(8):e0355721. doi: 10.1371/journal.pone.0355721 (PMC13466015; doi:10.1371/journal.pone.0355721)
Supplement: Supplementary file 3 [file pone.0355721.s005.docx]

**Response to reviewers**

We sincerely thank the Editor and the Reviewers for the time, effort, and thoughtful comments devoted to evaluating our manuscript. We greatly appreciate the constructive feedback, which has been invaluable in improving the scientific quality, clarity, and overall presentation of our work. We have carefully considered each comment and have revised the manuscript accordingly. Below, we provide a detailed, point-by-point response to every comment, indicating the changes made in the revised manuscript. We hope that the revisions satisfactorily address all concerns and that the manuscript is now suitable for publication in *PLOS ONE*

Journal requirements:

When submitting your revision, we need you to address these additional requirements.

1. Please ensure that your manuscript meets PLOS ONE's style requirements, including those for file naming. The PLOS ONE style templates can be found at

https://journals.plos.org/plosone/s/file?id=wjVg/PLOSOne_formatting_sample_main_body.pdf and

https://journals.plos.org/plosone/s/file?id=ba62/PLOSOne_formatting_sample_title_authors_affiliations.pdf

2. We note that there is identifying data in the Supporting Information file <23 cleaned DRTB_TREATMENT_OUTCOME_DATA_COLLECTION_TOOL_ET 2.xlsx>. Due to the inclusion of these potentially identifying data, we have removed this file from your file inventory. Prior to sharing human research participant data, authors should consult with an ethics committee to ensure data are shared in accordance with participant consent and all applicable local laws.

Data sharing should never compromise participant privacy. It is therefore not appropriate to publicly share personally identifiable data on human research participants. The following are examples of data that should not be shared:

-Name, initials, physical address

-Ages more specific than whole numbers

-Internet protocol (IP) address

-Specific dates (birth dates, death dates, examination dates, etc.)

-Contact information such as phone number or email address

-Location data

-ID numbers that seem specific (long numbers, include initials, titled “Hospital ID”) rather than random (small numbers in numerical order)

Data that are not directly identifying may also be inappropriate to share, as in combination they can become identifying. For example, data collected from a small group of participants, vulnerable populations, or private groups should not be shared if they involve indirect identifiers (such as sex, ethnicity, location, etc.) that may risk the identification of study participants.

Additional guidance on preparing raw data for publication can be found in our Data Policy (https://journals.plos.org/plosone/s/data-availability#loc-human-research-participant-data-and-other-sensitive-data) and in the following article: http://www.bmj.com/content/340/bmj.c181.long.

Please remove or anonymize all personal information, ensure that the data shared are in accordance with participant consent, and re-upload a fully anonymized data set. Please note that spreadsheet columns with personal information must be removed and not hidden as all hidden columns will appear in the published file.

If the reviewer comments include a recommendation to cite specific previously published works, please review and evaluate these publications to determine whether they are relevant and should be cited. There is no requirement to cite these works unless the editor has indicated otherwise.

**Response:** the data has been cleaned to meet journal specification

Additional Editor Comments:

Extensive revisions are required in methods, results, and discussion sections.

**Response**: thank you. The manuscript has been reviewed

Reviewer #1:

 This is a highly valuable, large-scale retrospective cohort analysis leveraging Zambia's national NTLP Yathu registry to uncover vital geographic and clinical disparities in MDR-TB care success.

However, before publication can be recommended, the following major and minor concerns must be resolved:
 **response:** Thank you. for your kind words

1.Introduction
Line 76: species name - write in italic

**Response:** thank you. it been corrected

Line 83-85: the text ‘while Bwembya
84 et al. (2024) found that 75% of TB-related deaths occurred within the first two months of treatment,85 predominantly among HIV-positive individuals’ change to research focusing on MDR TB.
 **Response**: We have revised Lines 83–85 by modifying the text for greater accuracy and replacing the previous citation with one that specifically reflects the epidemiology and outcomes of multidrug-resistant tuberculosis (MDR-TB) in Zambia.

2.Methods
Figure 1: Please re-upload or fix Figure 1 (the study flow diagram), as it fails to display clearly the research flow.

**Response**: thank you we have reuploaded a new flow diagram which is clear

Line 132: remove one post stop.

**Response:** thank you. post stop has been removed

Line 140: Operational definition
- How about weight? The weight at which the month of treatment was used?

**Response:** thank you for the observation we used the Weight at initiation of treatment we have now clarified
- Line 147–149: Mortality was defined as death from any cause occurring before or during the course of anti-tuberculosis treatment..."
Line 160–162: Death was defined as death from any cause before or during treatment..."
Operational definitions no2 (Mortality) and no4 (under Unfavorable outcomes) are entirely redundant and identical in substance. Please consolidate these into a single definition to streamline the Methods section.

**Response:** thank you. we have consolidated the definition as one.

Line 143: italic for species name ‘Mycobacterium tuberculosis”
**Response:** thank you . we have now italicized the species name

3.Results
The study aims (Line 98-99) stated to examine the % of MDR. However, no results were presented in the text.

**Response**: We thank the reviewer for this observation. The proportion of MDR-TB patients achieving favorable treatment outcomes (68.1%, n=857/1,258) was present in the manuscript but had not been explicitly foregrounded in the Results narrative as a direct answer to the stated primary aim. We have added a sentence at the opening of the Results section to clearly state this finding.

Line 206: capital T for Table 1

**Response:** thank you. we have corrected the wording

Table 1 & 2 & 3: in the last column, showing p-value. What analysis was used? In the text above, nothing was mentioned about the p-value. So, is the analysis necessary? The table should be presented scientifically, i.e., without visible lines for rows and columns, except the uppermost and lowermost. Standardise in writing p, italic or not, small or capital letter. For example, line 179 and line 182. 95CI should be written completely, 95% CI.

**Response:** We thank the reviewer for this observation. We have added a sentence to the Data Analysis section explicitly stating the tests used: "Differences in baseline characteristics between participants with favorable and unfavorable outcomes were assessed using the chi-square test for categorical variables and the Mann-Whitney U test for continuous variables (age)." We have retained the p-value columns in Tables 1 and 2 as they provide readers with a preliminary indication of which variables differ between outcome groups, supporting the biological plausibility of findings in the multivariable model. We have also reformatted all three tables in accordance with standard scientific journal style. Internal row and column gridlines have been removed. Only the topmost border, a thin rule beneath the column header row, and the bottommost border are retained. We have also standardised all instances of p throughout the manuscript text and tables to ***p*** (lowercase, italic), consistent with APA and most medical journal conventions. Inconsistent forms (P, P-value, p-value with capital P) have been corrected.

Line 221: Results of the regression analysis should include the model fitness as stated in lines 182-185.
***Response:*** We thank the reviewer for this observation. We agree that the model fitness statistics, although pre-specified in the Methods section, were not reported in the Results. We have now added them at the foot note of table three.

4.Discussion
Line 234: treatment success or favourable treatment? should be clear and standardised. Not only in this line, but throughout this manuscript.

**Response:** We thank the reviewer for this important observation. We have reviewed the manuscript and standardized the terminology by replacing all instances of "treatment success" with "favorable treatment outcome," which is the predefined primary outcome of this study and is consistent with the WHO and Zambia National Tuberculosis and Leprosy Programme outcome classification framework.

Line 246-247: should be cautious in interpreting the results from a cross-sectional study. The big limitation is that it cannot explain the causal effects of the factors. Thus, for the BMI, which one is the predictor? Poor treatment outcome causes low BMI, or Low BMI causes poor treatment outcome?

**Response:** We thank the reviewer for this important comment. We agree that, given the retrospective observational design of our study, causal relationships cannot be inferred. In particular, the association between low BMI and unfavorable treatment outcome should not be interpreted as causal, as reverse causality is possible; poor treatment outcomes may contribute to weight loss, while low BMI may also reflect underlying disease severity at treatment initiation. We have revised the Limitations section to explicitly acknowledge this limitation and have avoided causal language throughout the manuscript, replacing terms such as "predictor" with "associated factor" where appropriate.

Line 260-261: Elaborate on health care and support systems disparities among these provinces.

**Response:** We thank the reviewer for this valuable suggestion. We have expanded the Discussion to elaborate on the healthcare and support system disparities that may explain the observed provincial differences in treatment outcomes.

Line 288-289: also cautious on the interpretation since no information regarding Without knowing the exact proportion of PLHIV who were actively on ART, virally suppressed, or severely immunosuppressed (CD4 counts),
**Response:** We thank the reviewer for this important observation and agree that caution is warranted. As the dataset did not capture ART status, viral suppression, or CD4 cell count for the HIV-positive subgroup, we are unable to determine what proportion of participants living with HIV were actively on ART, virally suppressed, or severely immunosuppressed at treatment initiation. We have revised the Discussion to make this limitation explicit alongside our interpretation of the HIV co-infection finding, and we have reiterated it in the Limitations section, noting that the absence of these variables may have introduced residual confounding and should temper any causal interpretation of the null association observed between HIV status and treatment outcome.

Reviewer #2: General comment
The manuscript addresses a relevant and important topic; however, several methodological and reporting issues need to be resolved to strengthen the validity and interpretability of the findings.

Specific comments
As this is a retrospective cohort study, the authors should justify the use of logistic regression and odds ratios. Consider presenting risk ratios using log-binomial or modified Poisson regression models, particularly if the outcome is not rare.

**Response:** We thank the reviewer for this important observation. Our objective was to assess factors associated with favorable and unfavorable treatment outcomes using a cross-sectional analysis of routine programmatic data rather than a time-to-event approach. Accordingly, we have revised the manuscript to consistently reflect the cross-sectional nature of the study, avoided causal language, and expanded the Limitations section to acknowledge that causal relationships cannot be inferred. We also clarified that the absence of detailed HIV-related variables (e.g., ART status, viral suppression, and CD4 cell count) limits the interpretation of HIV-related findings.

The Methods section states that variables with p<0.20 in univariable analysis were entered into the multivariable model. However, variables such as age, smoking, previous history of TB, type of DRTB, TB classification, and TB regimen appear not to satisfy this criterion. Please clarify the variable selection strategy.
 **Response:** We thank the reviewer for this important observation. Although variables with **p < 0.20** in the univariable analysis were eligible for inclusion, some variables (e.g., age, smoking, previous history of TB, type of DR-TB, TB classification, and TB regimen) were also included **a priori** based on their established clinical and epidemiological relevance as potential confounders, irrespective of their univariable significance. We have revised the Methods section to clarify this two-step variable selection strategy

Since age was included as a continuous predictor in the logistic regression model, the assumption of linearity between age and the logit of the outcome should be assessed and reported. Please clarify whether this assumption was evaluated (e.g., using the Box–Tidwell test, fractional polynomials, restricted cubic splines, graphical assessment, or categorization of age where appropriate). If the linearity assumption was violated, alternative modeling approaches should be considered.

**Response:** We thank the reviewer for raising this important methodological point. Prior to multivariable modelling, we assessed the assumption of linearity between age and the log odds of the outcome using the Box–Tidwell test, which was non-significant (p = 0.137), indicating no evidence of departure from linearity. Age was therefore retained as a continuous linear term in the multivariable model. We have added this assessment, including the test used and the result, to the Methods (Statistical Analysis) section to make this explicit.

The p-value for the province variable is missing from Table 1.
**Response: thank you we have now included it**

Only 459 observations have BMI data, indicating substantial missingness. If BMI was included in the multivariable model, the effective sample size may have been considerably reduced. Please report the extent of missingness, the final sample size used in multivariable analyses, and the methods used to handle missing data.
**Response:** We thank the reviewer for this important observation. We have clarified the statistical methods in the Methods section and table footnotes. Specifically, we now state that p-values in **Tables 1 and 2** were obtained using Pearson's chi-square test or Fisher's exact test for categorical variables and the Mann–Whitney U test for continuous variables, as appropriate, whereas the p-values reported alongside the crude and adjusted odds ratios in **Table 3** were obtained from univariable and multivariable logistic regression, respectively.

In Table 4, the reported p-value for TB regimen is 1.21, which is not possible and should be checked.

**Response:** thank you for the correction. We have verified and correct it

Please specify the statistical tests used to generate p-values in Tables 1 and 2 and the p-values reported alongside crude ORs in Table 3. Several variables such as alcohol use and HIV status show markedly different p-values across tables (e.g., p-value for alcohol was 0.003 in Table 1 but p-value for alcohol in Table 3 was 0.117, similar issue for HIV status variable). Clarification regarding the statistical methods and interpretation is needed.
**Response:** We thank the reviewer for this observation and apologize for the lack of clarity. The p-values reported in Tables 1 and 2 were derived from the chi-square test for categorical variables and the Mann–Whitney U test for continuous variables (age), assessing unadjusted bivariate associations using all available observations for each variable. The p-values reported alongside the crude odds ratios (cORs) in Table 3, by contrast, were derived from univariable logistic regression restricted to the complete-case sample used in the multivariable model, since crude and adjusted estimates needed to be drawn from a consistent analytic sample for comparability. This difference in both statistical approach and underlying sample size explains the discrepancies the reviewer identified for alcohol use and HIV status. We have clarified this distinction in the Methods section and in the footnotes to Tables 1–3 to avoid this confusion for the reader.
Why was "Diabetes status" variable excluded in the multivariable model despite being significant in bivariate analysis?

**Response:** We thank the reviewer for this comment. Diabetes mellitus was excluded from the multivariable analysis because of its very low prevalence (14/1,250; 1.1%), which was insufficient to produce reliable adjusted estimates. We have clarified this in the manuscript and added it as a study limitation.

Based on the frequencies reported in the manuscript, I am unable to reproduce some crude odds ratios (e.g., HIV status) shown in Table 3. The authors should verify the analyses, reference categories, and reported estimates.

**Response:** We thank the reviewer for this important observation. We re-derived all crude and adjusted odds ratios in Table 3 directly from the underlying dataset to verify reproducibility. For HIV status specifically, recalculation confirms a crude OR of 1.10 (95% CI: 0.86–1.41, p = 0.437) using Negative as the reference category against the full available sample (n = 1,205), consistent with the bivariate result in Table 2. We have now specified reference categories explicitly in the Table 3 footnote for every variable, and we have independently re-verified each crude and adjusted estimate against the source data to confirm reproducibility.

Once again, we sincerely thank the Editor and the Reviewers for their constructive comments and valuable suggestions. We believe that these revisions have substantially strengthened the manuscript, and we hope that the revised version will now be suitable for publication in *PLOS ONE*.

Dr Siame Lukundo

On behalf of the authors
